# Influence of Interface Mixed Layer on Non-Collinear Exchange Coupling in V-Fe Multilayers

**DOI:** 10.3390/ma18030697

**Published:** 2025-02-05

**Authors:** Agnieszka Ranecka, Maria Pugaczowa-Michalska, Lesław Smardz

**Affiliations:** Institute of Molecular Physics, Polish Academy of Sciences, Smoluchowskiego 17, 60-179 Poznań, Poland; agnmar@ifmpan.poznan.pl (A.R.); maria@ifmpan.poznan.pl (M.P.-M.)

**Keywords:** magnetic thin films, hydrogen absorption, XPS, metallic multilayers

## Abstract

V/Fe multilayers were prepared on naturally oxidized Si(100) substrates at room temperature (RT) by UHV magnetron sputtering. Mixing effects at the Fe–V interfaces were investigated in-situ, directly after deposition, by means of X-ray photoelectron spectroscopy (XPS). The results of systematic in-situ XPS studies of the integral intensity of the Fe-2p peak, as a function of the nominal thickness of the Fe sublayer deposited on vanadium, allowed us to estimate the thickness of the pure iron layer that forms the mixed layer at about 0.4 nm. Assuming the same thickness of the vanadium layer that forms the mixed layer, the estimated thickness of the mixed layer near the Fe–V interface was about 0.8 nm. In the analysis of magnetic hysteresis loops, in addition to the bilinear (J_1_) and biquadratic (J_2_) coupling constant, the contribution of the cubic exchange constant (J_3_) was taken into account, which also contributed significantly to the total energy. Higher order interactions (J_2_ and J_3_) are particularly important for V spacer thicknesses greater than 7 atomic monolayers. Hydrogen absorption in V/Fe multilayers at RT and a pressure of about 1 bar causes an increase in the biquadratic coupling constant J_2_, while the values of J_1_ and J_3_ are reduced. A comparison of the obtained experimental results and available theoretical models leads to the conclusion that the mechanism of “fluctuating thickness of the non-magnetic spacer” could be responsible for the biquadratic exchange coupling. On the other hand, the “loose spins” model can explain the cubic coupling in the V/Fe multilayers. The modification of the interlayer exchange coupling using hydrogen is fully reversible.

## 1. Introduction

Short-ranged interlayer exchange coupling plays an important role in the properties and potential application of magnetic materials. However, the origin and nature of this interaction are not sufficiently clear [1]. The interlayer exchange coupling plays an important role in the characterization and potential application of magnetic multilayers (MLs) [2,3,4,5]. So far, the magnetic properties of many layered systems have been investigated, while the type and energy of interlayer exchange depending on the thickness of the non-magnetic sublayer have not been fully elucidated. The interlayer exchange coupling oscillates between ferro- and antiferromagnetic ordering with the thickness of the non-magnetic spacer. The experimental periods of oscillation are of the order of 1 nm [6] and are in agreement with the theory based on the Ruderman–Kittel–Kasuya–Yosida (RKKY) interaction [7], and could also be alternatively described by quantum wells in the spacer layer [8].

The great interest in the antiferromagnetic coupling of the iron sublayers across the non-magnetic spacer of vanadium resulted from the possibility of modifying this coupling by the absorption of hydrogen in V [9] or Nb [10,11] spacers. The iron and vanadium layers have the same bcc-type crystallographic structure and relatively small differences in the lattice parameters [9]. Experimental studies of hysteresis loops of magnetic multilayers have so far shown that their description should usually take into account bilinear and biquadratic coupling (i.e., 90° coupling). Effective interaction is the result of competition between different types of interactions [12,13]. Until now, the theoretical description of the interlayer coupling was limited to two terms: bilinear and biquadratic [14,15,16,17,18,19].

Furthermore, in theoretical papers [16,17,18], it has been shown that roughness and interdiffusion at the interface contribute to the formation of significant biquadratic coupling. In Ref. [20], it has been experimentally confirmed that a large roughness parameter at the interface leads to the formation of significant interlayer biquadratic coupling in Ni_80_Fe_20_/Cr/Ni_80_Fe_20_ trilayers. Moreover, the effect of interface roughness on the magnetization reversal process in Co/Cu multilayers has been studied quite recently [21].

In this contribution, we report results on the interface characterization and higher order interlayer coupling studies in V/Fe multilayers. The aim of this work is to investigate and determine the mechanisms leading to non-collinear interlayer exchange coupling in V/Fe MLs.

## 2. Experimental Procedure

V/Fe MLs were deposited onto naturally oxidized Si(100) substrates at room temperature (RT) [22,23,24] by ultra-high vacuum (UHV) magnetron sputtering under a high-purity argon atmosphere. The DC (direct current) mode was used for the preparation of iron and palladium layers, while the vanadium layers were deposited using the RF (radio frequency) mode. More details concerning preparation conditions can be found in Refs. [22,23,24].

**Figure 1:**
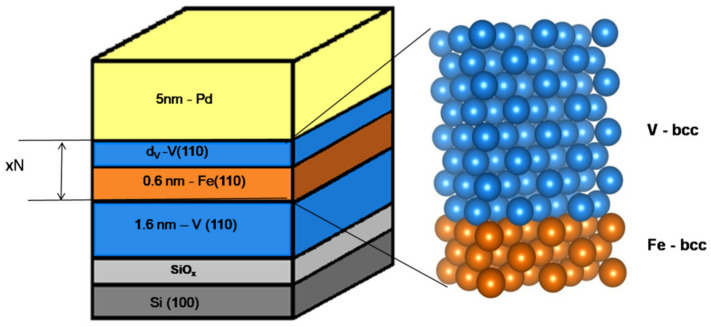
Schematic representation of the studied V/Fe multilayers.

The assumed sequence of the deposited multilayers is shown in Figure 1. Before preparing the V/Fe MLs, a 1.6 nm thick V buffer layer was first deposited, which corresponds to approximately 8 monolayers (ML) of V(110). The thickness of the Fe sublayer was constant and was about 0.6 nm (about 3 ML of Fe(110)). The number of repetitions of the basic period varied from 25 to 100. In addition, a protective palladium layer with a thickness of 5 nm was used to protect the iron and vanadium layers from oxidation in air. This layer also acts as a catalyst in the process of hydrogen absorption and desorption. The chemical composition and the width of the interfaces of the deposited MLs were determined in-situ, using X-ray photoelectron spectroscopy (XPS) with Mg-Kα radiation. More details on XPS measurements can be found in Ref. [24]. Standard X-ray diffraction (XRD) revealed the strong (110) texture of the prepared multilayers [22]. The magnetic properties were measured with a vibrating sample magnetometer at RT in a magnetic field up to ±9 T.

In our ab initio calculation, the V/Fe MLs were modelled as periodic structures of Fe-3ML/V-nML/Fe-3ML layers stacked along the (110) direction with ferromagnetic and antiferromagnetic coupled magnetic slabs of Fe. The calculations were carried out using the projector augmented wave (PAW method) [25] implementation of the Density Functional Theory (DFT) method of pseudopotentials—VASP code [26,27]. In the first step, we constructed supercell models between bcc-Fe and bcc-V (that are both stacked along the (110) direction) with an initial in-plane lattice parameter of 4.05879 Å and a variable out-of-plane lattice parameter corresponding to the monolayer number of vanadium (i.e., V-n ML). Such supercells were relaxed according to the procedures in the VASP code. For all the supercells, a plane wave energy cut-off of 400 eV, a total energy convergence threshold of 10^−6^ eV, and a force stopping criterion of 0.01 eV/Å were used. The exchange–correlation energy was chosen in the generalized gradient approximation (GGA) [28]. The calculation method is described in more detail in Ref. [23].

The V/Fe MLs (see Figure 1) were hydrogenated in a high-vacuum chamber up to ~1 bar at RT for at least one hour. Hydrogen absorption can only occur in the V sublayers. The Fe sublayers do not absorb hydrogen at all. Hydrogen desorption occurs spontaneously after the sample’s exposure to air.

## 3. Results and Discussion

**Figure 2:**
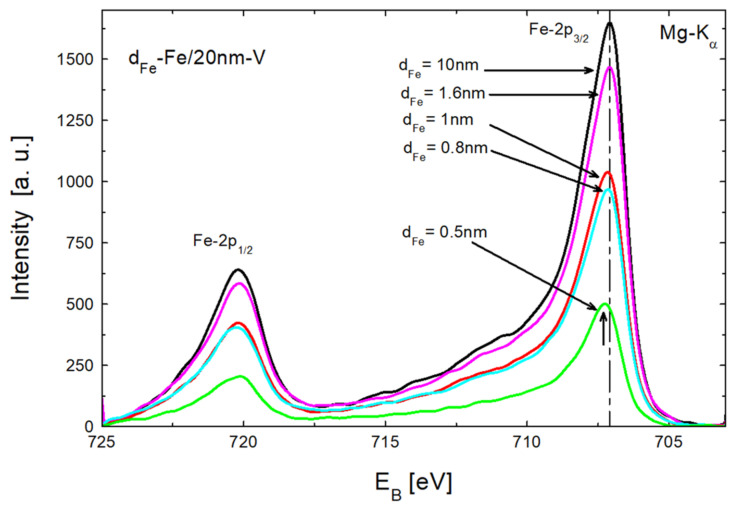
Fe-2p XPS spectra measured for d_Fe_—Fe/20 nm—V bilayers with different Fe thicknesses. The position of Fe-2p_3/2_ peak measured for the 20 nm—Fe reference layer is denoted by a vertical broken line.

Layered structures of all prepared Fe/V MLs were revealed in the X-ray diffraction studies. We observed intense satellite reflection in the high- and low-angle XRD patterns, as already reported in Ref. [22]. Furthermore, the samples were deposited at RT and therefore the interdiffusion at interfaces should be limited. The sharp interface is especially relevant in the case of magnetic MLs. For instance, the formation of magnetic bridges in the area of the non-magnetic layer leads to a lack of antiferromagnetic coupling. In order to estimate the width of the mixed layer at the Fe-V interface, we performed systematic XPS studies of the Fe-2p peak for different Fe thicknesses, similarly to the previous studies of the mixed layer for Pd-Gd, Pd-Ni, Ni-Gd [29], Pd-Y, Pd-Ti, and Ti-Y interfaces [30]. Figure 2 shows Fe-2p spectra measured in-situ for freshly prepared Fe/V bilayers with Fe layer thicknesses varying from 0.5 nm to 10 nm. The thickness of the vanadium layer was constant at 20 nm. The position of the Fe-2p_3/2_ peak measured for d_Fe_ = 0.5 and 1 nm is shifted with respect to the position measured for the reference 20 nm—Fe layer (vertical broken line). The shift is mainly caused by interdiffusion at the Fe-V interface during the growth in the deposition process. The thickness of the mixed layer is strongly limited; therefore, the d_Fe_ = 1.6 nm position of the Fe-2p_3/2_ peak is close to the position observed for pure iron (vertical broken line). Moreover, the centroids of all Fe-2p peaks are shifted relative to the reference layer towards higher energies. Due to the significantly higher integral intensity of the Fe-2p_3/2_ peaks and the associated higher measurement accuracy, the data of these peaks will be used for further studies.

**Figure 3:**
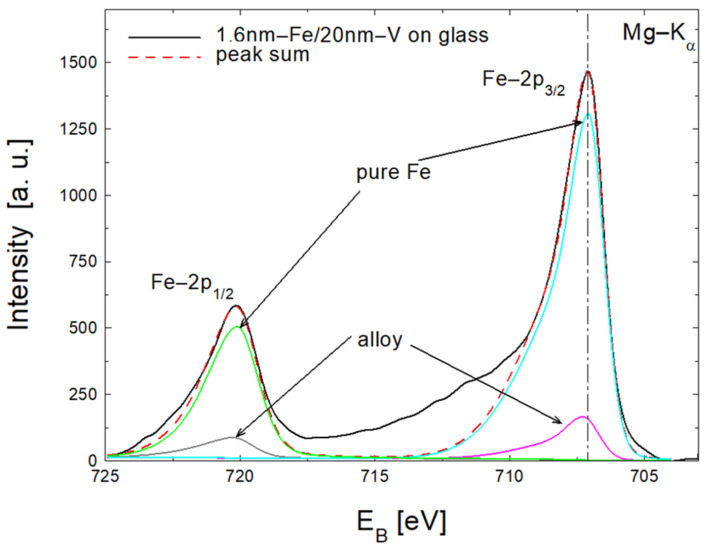
Deconvolution of Fe-2p peaks to XPS signals from the ultra-thin mixed (alloy) Fe-V layer and pure iron. The sums of the deconvoluted peaks are marked with red dotted lines.

In Figure 3, we show an example deconvolution of the Fe-2p spectrum measured for 1.6 nm Fe/20 nm V bilayer into the signal originated from the ultra-thin Fe-V alloy layer and pure Fe layer. The pure Fe layer is located on top of the sample. The mixed Fe-V layer with variable Fe concentrations is located in the interface area. A pure V layer is located below the mixed Fe-V layer. For the 1.6 nm Fe, the Fe-2p peak from the mixed Fe-V layer is smaller compared to the peak fitted for the pure Fe layer (Figure 3). The sums of two fitted peaks are represented by a broken line. The locations of the experimental (solid line) and fitted (broken line) Fe-2p peaks are exactly the same. The Fe concentration in the mixed layer in the direction perpendicular to the substrate is variable. The concentration of Fe atoms near the pure V layer is low, while being simultaneously high near the pure iron layer. Considering the XPS signal of the pure iron layer from the top part of the nominally deposited Fe, and the Fe-2p signal detected from the mixed layer located just below, the thickness of the lost nominally deposited Fe layer should be smaller than the thickness of the top pure Fe layer. If we increase the thickness of the deposited iron to 10 nm, we no longer observe any signal from the mixed layer due to the limited information depth of the XPS method.

Based on the XPS theory [31], and denoting the thickness of the Fe layer forming the mixed layer as d_m_ and the thickness of the pure iron layer as d = d_Fe_ − d_m_, the integral intensities for 2p_3/2_ peaks (I) of the top pure Fe layer can be described as follows [29,30]:

I = I_0_(1 − e^−d/L^) (1)

**Figure 4:**
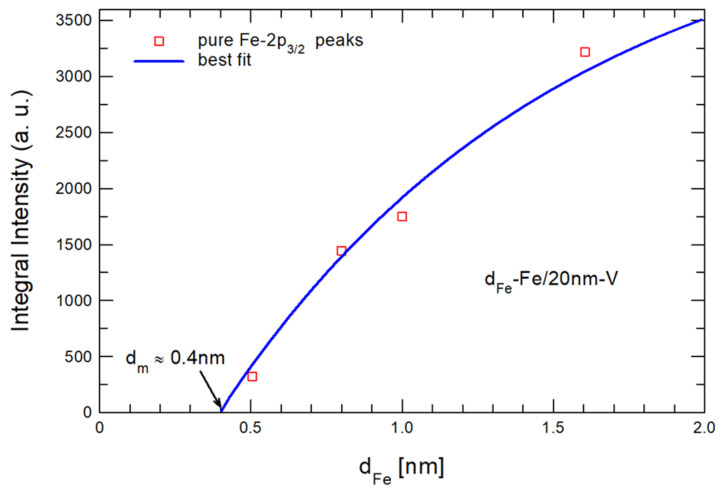
Integral intensities of fitted pure Fe-2p_3/2_ peaks as a function of nominal (deposited) Fe thickness for d_Fe_—Fe/20 nm—V bilayers. The lost thickness of the deposited pure iron layer, which forms the mixed Fe-V layer, was estimated as d_m_~0.4 nm.

L denotes the escape depth of the excited photoelectrons (L = 1.15 nm for Fe-2p [31]). In Figure 4, we show the integral intensities of the fitted pure Fe-2p_3/2_ peaks (see Figure 3) as a function of the nominal (deposited) iron thickness, d_Fe_. The thickness dependence of the integral intensities (squares) was fitted using Equation (1). The best fit, denoted by the solid line in Figure 4, was obtained for I_0_ = 38,000 a.u. and L = 1.15 nm. The lost thickness of the deposited pure iron layer, which forms the mixed Fe-V layer, was estimated as d_m_~0.4 nm. Assuming the same thickness of the vanadium layer that forms the mixed layer, the estimated thickness of the mixed layer near the Fe–V interface was about 0.8 nm.

**Figure 5:**
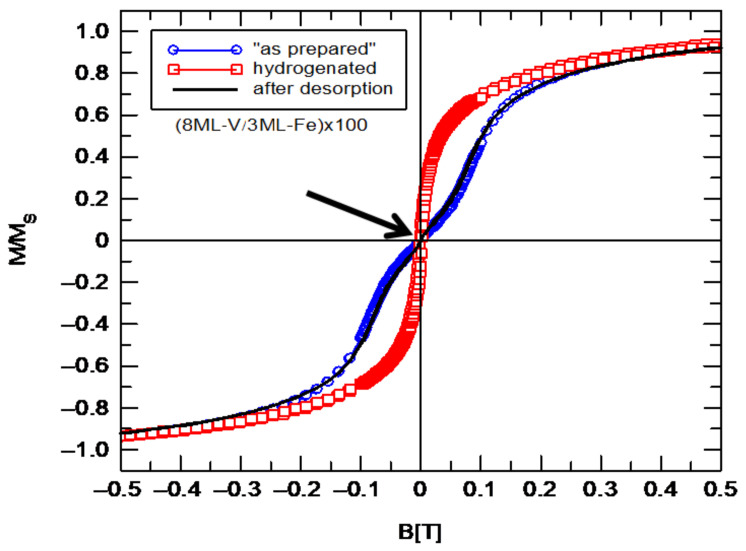
Hysteresis loops of (3ML-Fe/8ML-V) × 100 multilayer measured at RT before (blue circles) and after hydrogen absorption (red squares) and desorption (black line). The black arrow indicates the characteristic “inflection point” of the hysteresis loop.

The mixed layer, located between pure Fe and V layers, is very important in exchange coupling propagation. In Figure 5, we show hysteresis loops measured for (8ML-V/3ML-Fe) × 100 multilayer (blue circles). For the “as prepared” sample, a characteristic “inflection” near zero can be observed (Figure 5). This “inflection” of the hysteresis loop disappears after hydrogen absorption, as shown in Figure 5 (red squares). After 24 h of hydrogen desorption in air for the sample, the corresponding hysteresis loop (black line in Figure 5) is identical to the hysteresis loop measured for the “as prepared” sample (blue circles). This indicates that the modification of the hysteresis loop using hydrogen is fully reversible. The inset in Figure 5 shows the hysteresis loops before and after hydrogen absorption measured in a magnetic field up to 1.2 T.

**Figure 6:**
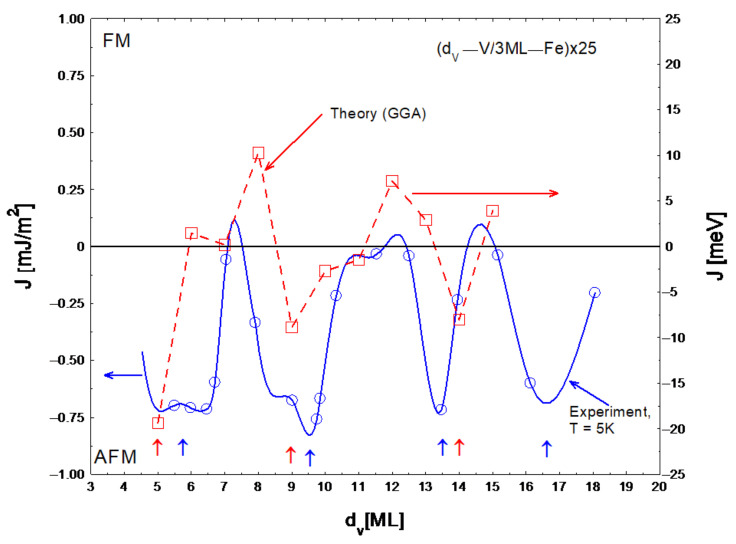
Interlayer exchange coupling energy as a function of vanadium layer thickness: experiment (blue circles) and theory (red squares). The dashed and solid lines connecting the symbols are a guide for the eye. Blue (experiment) and red (theory) vertical arrows indicate the minima of the coupling energy. The red squares correspond to the calculated points for supercells in which the atoms are fixed at their crystallographic positions according to DFT. The horizontal arrows indicate the proper scale.

The saturation magnetization M_s_ of the Fe sublayers was strongly reduced, mainly due to the alloying effect at interfaces and d_Fe_ = 3 ML was measured at 5 K as 1:1 T. The interlayer exchange coupling per unit surface J is equal to [23] J = (1/4)M_s_μ_o_H_s_d_Fe_. Figure 6 shows interlayer exchange coupling energy as a function of vanadium layer thickness, with the experiment at T = 5 K (blue circles) and ab initio calculations with GGA approximation [23] (red squares). 

Considering that the experiment based on hysteresis loop measurements does not allow for determining the ferromagnetic coupling energy, the qualitative agreement of the experiment with the theory is good. The minima of the J(d_v_) dependence correspond to the maxima of the antiferromagnetic exchange interaction. It should be emphasized that the ab initio calculations were performed for an ideal structure (single crystal) without taking into account the alloying effect at the interfaces [23]. In the real V/Fe MLs, there is an alloying effect and non-zero roughness at the interfaces, which can consequently lead to a shift of the antiferromagnetic coupling peaks. Additionally, in the interface regions, a magnetic moment is induced on vanadium atoms as a result of the proximity effect with iron atoms [23]. The proximity effect can be responsible for the absence of the antiferromagnetic coupling for the ultra-thin V spacer [23,32]. According to the positions of the vertical arrows in Figure 6, there is sufficient agreement with the theory of the oscillation period of the antiferromagnetic coupling, which is about 0.8 nm (4 ML). Moreover, the determined oscillation period of 0.8 nm for our (110) oriented MLs is consistent with the period determined in Ref. [32] for the (100)V/(110)Fe superlattices.

The experimental studies carried out so far on the hysteresis loops of multilayer ferromagnetic/non-magnetic systems have shown that the Stoner–Wohlfarth model can be used to describe them. The configuration of the magnetization vectors of adjacent ferromagnetic layers is the result of the competition of different kinds of magnetic interactions. So far, in the theoretical description, the exchange coupling has been limited to two terms: bilinear and biquadratic [14,15,16,17,18,19,20]. In this work, we additionally take into account the contribution from the cubic constant of exchange coupling, which may also contribute to the total energy.

After adding the cubic term (J_3_), the total energy of interaction of the coupled multilayer can be written as follows:

(2) $$E_{\mathrm{Tot}}={-J}_{1}\cos∆\varphi -J_{2}\cos^{2}∆{\varphi -J}_{3}\cos^{3}∆{\varphi -2d}_{\mathrm{Fe}}M_{\mathrm{Fe}}\mu_{0}H\;\cos∆{\varphi -\mathrm{Kcos}}^{2}∆\varphi$$

where

J_1_, J_2_, and J_3_ denote bilinear, biquadratic, and cubic exchange constants, respectively;
M_Fe_—magnetization of the Fe layer;
d_Fe_—iron layer thickness;
Δφ—angle between the magnetization vectors of adjacent Fe layers;
H—external magnetic field;
μ_0_—magnetic permeability in vacuum; and
K—uniaxial anisotropy constant.

**Figure 7:**
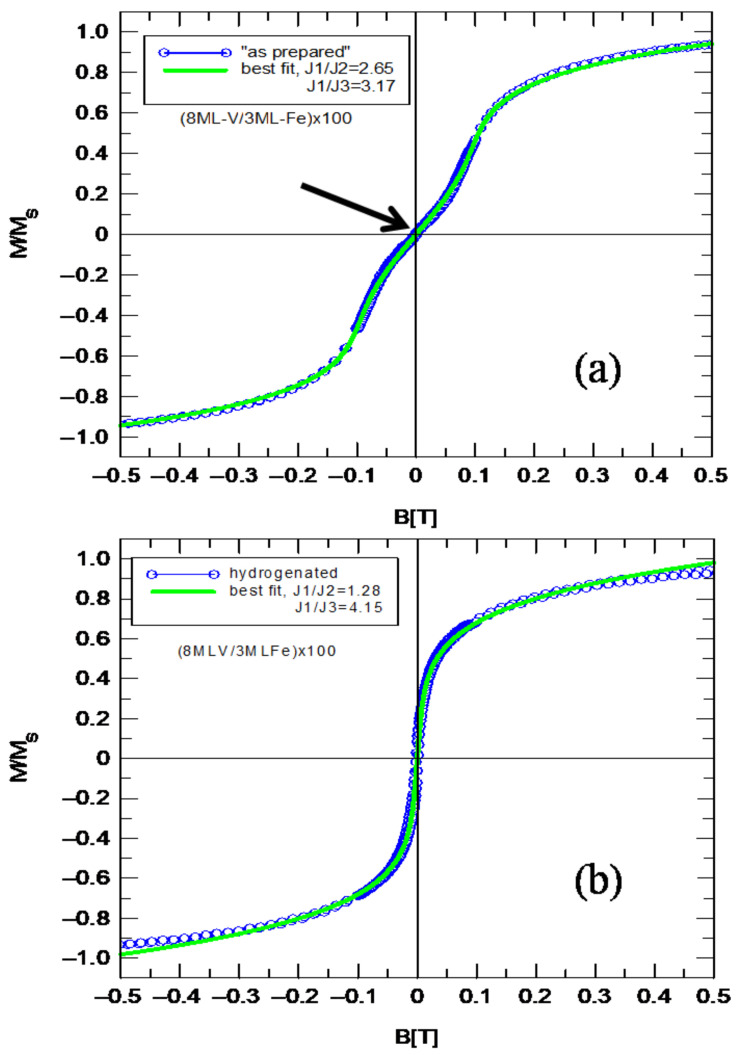
Hysteresis loop (blue circles) measured for (8ML-V/3ML-Fe) multilayer at RT before (**a**) and after (**b**) hydrogen absorption. Solid green line represents the best fit using bilinear (J_1_), biquadratic (J_2_) and cubic (J_3_) exchange constants.

The Stoner–Wohlfarth model has been adapted to simulate hysteresis loops. The examined multilayers practically did not show anisotropy in the plane; therefore, to simplify the simulation of the magnetization curve, the term related to the anisotropy was omitted from Equation (2). The hysteresis loop shown in Figure 7a revealed the antiparallel alignment of the magnetic moments of the neighboring Fe sublayers, fitted using bilinear (J_1_), biquadratic (J_2_) and cubic (J_3_) exchange constants. The best fit is presented in Figure 7a,b (green solid line) for “as prepared” and hydrogenated samples, respectively. The best fit (green solid line) for the “as prepared” sample was obtained for the coupling constants J_1_/J_2_ = 2.65 and J_1_/J_3_ = 3.17 giving J_2_/J_3_ = 1.19 (Figure 7a). After the absorption of hydrogen, there is a clear increase in J_2_ and a decrease in J_3_, giving J_2_/J_3_ = 3.24 (Figure 7b). The hysteresis loop practically showed small characteristic inflections after hydrogen absorption, for which we believe the cubic constant (J_3_) of the interlayer exchange coupling is mainly responsible. The higher-order interactions (J_2_ and J_3_) are especially important for V spacers containing more than seven monolayers. After hydrogen absorption, the shape of the hysteresis loop is changed, and thus the magnetic response of the sample also changes. This is related to the noncollinear arrangement of magnetic moments of adjacent Fe sublayers. The strong “inflection” at H = 0 can be seen for the hysteresis loop only when the bilinear coupling is weak enough. For V/Fe MLs, before hydrogen absorption, the bilinear coupling constant (J_1_) is much greater than the biquadratic (J_2_) and cubic (J_3_) coupling constants. The hydrogenation process is completely reversible and the layer returns to its original state after the natural desorption of hydrogen in the air.

Hydrogen uptake, in a clean layer of vanadium at RT, to saturation takes place in about 3 min. Also, the desorption of hydrogen in air at room temperature takes place in up to 10 min. On the other hand, the absorption of hydrogen in ultra-thin V-Fe alloys takes place within 2 h, and the complete desorption of hydrogen occurs only after about 24 h. As shown earlier, from the theoretical fit of the XPS signal to the obtained experimental data, the thickness of the mixed layer was determined to be about 0.8 nm for V/Fe MLs. Considering the constant nominal thickness of the Fe sublayer in V/Fe MLs of 0.6 nm, the ferromagnetic sublayer is in fact formed by the Fe-V alloy with variable iron concentration. In general, hydrogen absorption can also modify the interfaces, increasing their roughness and introducing additional stresses. Taking into account Slonczewski models of the mechanisms responsible for the formation of biquadratic exchange coupling [16,17,18], we postulate the introduction of cubic coupling in order to better fit the experimental hysteresis loop. The results presented in Figure 7a,b and Slonczewski’s models show that the biquadratic coupling in the weakly coupled V/Fe MLs could be caused by fluctuations in the effective thickness of the non-magnetic spacer. Hydrogen absorption in the mixed layer leads to an increase in the thickness fluctuation of the non-magnetic spacer, and thus to an increase in the energy of the biquadratic coupling. On the other hand, the absorption of hydrogen in the mixed layer leads to the formation of non-magnetic hydrides of ultra-thin Fe-V layers, which could lead to the disappearance of “loose spins”. As a consequence, hydrogenation causes the deactivation of the “loose spins” mechanism, which is probably responsible for the occurrence of relatively large cubic coupling. The hydrogenation process is fully reversible and repeatable. After the complete desorption of hydrogen from the mixed V-Fe layer, there is a return to the initial (before hydrogenation) hysteresis loop (Figure 7a).

## 4. Conclusions

The hydrogenation of weakly coupled V/Fe multilayers at RT and under pressure up to 1 bar causes an increase in the biquadratic coupling constant J_2_, while the values of J_1_ and J_3_ decrease. A comparison of the obtained experimental results and the theoretical models by Slonczewski [16,17,18] leads to the conclusion that the mechanism of “fluctuating thickness of the non-magnetic spacer” is responsible for the biquadratic exchange coupling. On the other hand, the “loose spins” model could explain the occurrence of cubic coupling in the V/Fe multilayers. Modification of the interlayer exchange coupling through hydrogen absorption is fully reversible. After the desorption of hydrogen in air at RT, all the samples returned to their original (“as prepared”) state.

## Data Availability

The original contributions presented in this study are included in the article. Further inquiries can be directed to the corresponding author.
